# Supplementary material for: Infection prevention and control compliance among exposed healthcare workers in COVID-19 treatment centers in Ghana: A descriptive cross-sectional study
Source: PLoS One. 2021 Mar 9;16(3):e0248282. doi: 10.1371/journal.pone.0248282 (PMC7943010; doi:10.1371/journal.pone.0248282)
Supplement: S1 Table — (DOCX) [file pone.0248282.s001.docx]

**S1 Table: Adherence to infection prevention and control procedures during health care interactions**

| **Variables** | **Frequency** | **Percent** |
| --- | --- | --- |
| **PPE USE Domain** |  |  |
| **Single-use gloves** |  |  |
| Always, as recommended | 303 | 92.4 |
| Most of the time | 14 | 4.3 |
| Occasionally | 6 | 1.8 |
| Rarely | 5 | 1.5 |
| Total | 328 | 100.0 |
| **Medical mask** |  |  |
| Always, as recommended | 300 | 91.5 |
| Most of the time | 24 | 7.3 |
| Occasionally | 2 | 0.6 |
| Rarely | 2 | 0.6 |
| Total | 328 | 100.0 |
| **Face shield or goggles/protective glass** |  |  |
| Always, as recommended | 284 | 86.6 |
| Most of the time | 30 | 9.2 |
| Occasionally | 9 | 2.7 |
| Rarely | 5 | 1.5 |
| Total | 328 | 100.0 |
| **Disposal gown** |  |  |
| Always, as recommended | 297 | 90.6 |
| Most of the time | 11 | 3.4 |
| Occasionally | 4 | 1.2 |
| Rarely | 16 | 4.9 |
| Total | 328 | 100.0 |
| During a health care interaction with the COVID-19 patient, did you remove and replace your PPE according to protocol (e.g. when medical mask became wet, disposed the wet PPE in the waste bin, performed hand hygiene, etc.)? |  |  |
| Always, as recommended | 303 | 92.4 |
| Most of the time | 19 | 5.8 |
| Occasionally | 1 | 0.3 |
| Rarely | 5 | 1.5 |
| Total | 328 | 100.0 |
| **Hand hygiene Domain** |  |  |
| During a health care interaction with the COVID-19 patient, did you perform hand hygiene before and after touching the COVID-19 patient (whether or not you were wearing gloves)? |  |  |
| Always, as recommended | 310 | 94.5 |
| Most of the time | 9 | 2.7 |
| Occasionally | 5 | 1.5 |
| Rarely | 4 | 1.2 |
| Total | 328 | 100.0 |
| During a health care interaction with the COVID-19 patient, did you perform hand hygiene before and after any clean or aseptic procedure was performed (e.g. while inserting a peripheral vascular catheter, urinary catheter, intubation, etc.)? |  |  |
| Always, as recommended | 303 | 92.4 |
| Most of the time | 5 | 1.5 |
| Occasionally | 14 | 4.3 |
| Rarely | 6 | 1.8 |
| Total | 328 | 100.0 |
| During a health care interaction with the COVID-19 patient, did you perform hand hygiene after exposure to body fluid? |  |  |
| Always, as recommended | 302 | 92.1 |
| Most of the time | 10 | 3.1 |
| Occasionally | 10 | 3.1 |
| Rarely | 6 | 1.8 |
| Total | 328 | 100.0 |
| During a health care interaction with the COVID-19 patient, did you perform hand hygiene after touching the patient’s surroundings (bed, door handle, etc.), regardless of whether you were wearing gloves? |  |  |
| Always, as recommended | 316 | 96.3 |
| Most of the time | 6 | 1.8 |
| Occasionally | 4 | 1.2 |
| Rarely | 2 | 0.6 |
| Total | 328 | 100.0 |
| During a health care interaction with the COVID-19 patient, were high touch surfaces decontaminated frequently (at least three times daily)? |  |  |
| Always, as recommended | 301 | 91.8 |
| Most of the time | 18 | 5.5 |
| Occasionally | 3 | 0.9 |
| Rarely | 6 | 1.8 |
| Total | 328 | 100.0 |
