# Supplementary material for: Infection prevention and control compliance among exposed healthcare workers in COVID-19 treatment centers in Ghana: A descriptive cross-sectional study
Source: PLoS One. 2021 Mar 9;16(3):e0248282. doi: 10.1371/journal.pone.0248282 (PMC7943010; doi:10.1371/journal.pone.0248282)
Supplement: S2 Table — (DOCX) [file pone.0248282.s002.docx]

**S2 Table: Adherence to Infection prevention and control measures when performing aerosol-generating procedures**

| **Variables** | Frequency | Percent |
| --- | --- | --- |
| **PPE usage Domain** |  |  |
| **Single-use gloves** |  |  |
| Always, as recommended | 80 | 100.0 |
| Most of the time | 0 | 0.0 |
| Occasionally | 0 | 0.0 |
| Rarely | 0 | 0.0 |
| Total | 80 | 100.0 |
| **N95 Respirator or equivalent** |  |  |
| Always, as recommended | 74 | 92.5 |
| Most of the time | 6 | 7.5 |
| Occasionally | 0 | 0.0 |
| Rarely | 0 | 0.0 |
| Total | 80 | 100.0 |
| **Face shield or goggles/protective glass** |  |  |
| Always, as recommended | 74 | 92.5 |
| Most of the time | 6 | 7.5 |
| Occasionally | 0 | 0.0 |
| Rarely | 0 | 0.0 |
| Total | 80 | 100.0 |
| **Disposal gown** |  |  |
| Always, as recommended | 79 | 98.8 |
| Most of the time | 1 | 1.3 |
| Occasionally | 0 | 0.0 |
| Rarely | 0 | 0.0 |
| Total | 80 | 100.0 |
| During aerosol-generating procedures on the COVID-19 patient, did you remove and replace your PPE according to protocol (e.g. when medical mask became wet, disposed the wet PPE in the waste bin, performed hand hygiene, etc.)? |  |  |
| Always, as recommended | 73 | 91.3 |
| Most of the time | 5 | 6.3 |
| Occasionally | 1 | 1.3 |
| Rarely | 1 | 1.3 |
| Total | 80 | 100.0 |
| **Hand hygiene domain** |  |  |
| During aerosol-generating procedures on the COVID-19 patient, did you perform hand hygiene before and after touching the COVID-19 patient, regardless of whether you were wearing gloves? |  |  |
| Always, as recommended | 79 | 98.8 |
| Most of the time | 1 | 1.3 |
| Occasionally | 0 | 0.0 |
| Rarely | 0 | 0.0 |
| Total | 80 | 100.0 |
| During aerosol-generating procedures on the COVID-19 patient, did you perform hand hygiene before and after any clean or aseptic procedure was performed? |  |  |
| Always, as recommended | 79 | 98.8 |
| Most of the time | 1 | 1.3 |
| Occasionally | 0 | 0.0 |
| Rarely | 0 | 0.0 |
| Total | 80 | 100.0 |
| During aerosol-generating procedures on the COVID-19 patient, did you perform hand hygiene after exposure to body fluid? |  |  |
| Always, as recommended | 73 | 91.3 |
| Most of the time | 6 | 7.5 |
| Occasionally | 1 | 1.3 |
| Rarely | 0 | 0.0 |
| Total | 80 | 100.0 |
| During aerosol-generating procedures on the COVID-19 patient, did you perform hand hygiene after touching the patient’s surroundings (bed, door handle, etc), regardless of whether you were wearing gloves? |  |  |
| Always, as recommended | 80 | 100.0 |
| Most of the time | 0 | 0.0 |
| Occasionally | 0 | 0.0 |
| Rarely | 0 | 0.0 |
| Total | 80 | 100.0 |
| During aerosol-generating procedures on the COVID-19 patient, were high-touch surfaces decontaminated frequently (at least three times daily) |  |  |
| Always, as recommended | 73 | 91.3 |
| Most of the time | 6 | 7.5 |
| Occasionally | 1 | 1.3 |
| Rarely | 0 | 0.0 |
| Total | 80 | 100.0 |
