# Supplementary material for: Infection prevention and control compliance among exposed healthcare workers in COVID-19 treatment centers in Ghana: A descriptive cross-sectional study
Source: PLoS One. 2021 Mar 9;16(3):e0248282. doi: 10.1371/journal.pone.0248282 (PMC7943010; doi:10.1371/journal.pone.0248282)
Supplement: S1 File — Questionnaire used for data collection. (DOCX) [file pone.0248282.s003.docx]

**S1 File: Questionnaire for Infection prevention and control compliance among exposed healthcare workers in COVID-19 treatment centers in Ghana: a descriptive cross-sectional study**

**English questionnaire**

**Respondent Identification Code……………. Region of Respondent………………….**

|  | **Section A: Healthcare worker characteristics and availability of IPC facilities** |  |
| --- | --- | --- |
|  |  |  |
| Q1 | Age in years |  |
| Q2 | Gender | 1, Male  2, Female |
| Q3 | Marital status | 1, Married  2, Single  3, Separated/divorced  4. Widowed |
| Q4 | Highest qualification | 1, Basic education  2, Secondary level qualification  3, Certificate  4, Diploma  5, Bachelors  6, Masters |
| Q5 | Type of health professionals | 1, Assistant nurse  2, Cleaner  3, Laboratory personnel  4, Medical doctor  5, Registered nurse  6, Pharmacist  7, Physical and Respiratory Therapist  8, Clinical engineer  9, Admission and reception clerk  10, Administrative staff  12, IT personnel |
| Q6 | How many years have you been working | ………………… |
| Q7 | In the past one week, have you experienced an interruption in water supply in this facility? | 1, Yes  2, No |
| Q8 | Is PPE available in sufficient quantity in these healthcare facilities? | 1, Yes  2, No |
| Q9 | Have you ever received training on infection prevention and control? | 1, Yes  1, No |
|  | **Section B: Healthcare worker activities performed on COVID-19 patient in health care facility** | |
| Q10 | Did you provide direct care to a confirmed COVID-19 patient? | 1, Yes  2, No  3, Unknown |
| Q11 | Did you have face-to-face contact (within 1 metre) with a confirmed COVID-19 patient in a health care facility? | 1, Yes  2, No  3, Unknown |
| Q12 | Were you present when any aerosol-generating procedures were performed on the patient? | 1, Yes,  2, No  3, Unknown |
| Q13 | Did you have direct contact with the environment where the confirmed COVID-19 patient was cared for? E.g., bed, linen, medical equipment, bathroom etc | 1, Yes  2, No  3, Unknown |
|  | **NB:** *If the health worker responds ‘Yes’ to any of the Questions Q10 – Q13 the health worker should be considered as being exposed to COVID-19 virus.* *Qualified to proceed to answer questions on IPC adherence during healthcare interactions with COVID-19 patients.* |  |
|  | **Section C: Adherence to IPC procedures during health care interactions** | |
|  | *For the following questions, please quantify the frequency with which you wore PPE, as recommended: ‘Always, as recommended’ means more than 95% of the time; ‘Most of the time’ means 50% or more but not 100%; ‘occasionally’ means 20% to under 50% and ‘Rarely’ means less than 20%.* | |
| Q14 | During a health care interaction with a COVID-19 patient, did you wear personal protective equipment (PPE)? | 1, Yes  2, No |
|  | **If yes, for each item of PPE below, indicate how often you used it?** |  |
|  | Single-use gloves | 1, Always, as recommended  2, Most of the time  3 Occasionally  4, Rarely |
|  | N95 mask (or equivalent respirator) | 1, Always, as recommended  2, Most of the time  3, Occasionally  4, Rarely |
|  | Face shield or goggles/protective glasses | 1, Always, as recommended  2, Most of the time  3, Occasionally  4, Rarely |
|  | Disposable gown | 1, Always, as recommended  2, Most of the time  3, Occasionally  4, Rarely |
| Q15 | During a health care interaction with the COVID-19 patient, did you remove and replace your PPE according to protocol (e.g. when medical mask became wet, disposed the wet PPE in the waste bin, performed hand hygiene, etc.)? | 1, Always, as recommended  2, Most of the time  3, Occasionally  4, Rarely |
| Q16 | During a health care interaction with the COVID-19 patient, did you perform hand hygiene before and after touching the COVID-19 patient (whether you were wearing gloves)? | 1, Always, as recommended  2, Most of the time  3, Occasionally  4, Rarely |
| Q17 | During a health care interaction with the COVID-19 patient, did you perform hand hygiene before and after any clean or aseptic procedure was performed (e.g. while inserting a peripheral vascular catheter, urinary catheter, intubation, etc.)? | 1, Always, as recommended  2, Most of the time  3, Occasionally  4, Rarely |
| Q18 | During a health care interaction with the COVID-19 patient, did you perform hand hygiene after exposure to body fluid? | 1, Always, as recommended  2, Most of the time  3, Occasionally  4, Rarely |
| Q19 | During a health care interaction with the COVID-19 patient, did you perform hand hygiene after touching the patient’s surroundings (bed, door handle, etc.), regardless of whether you were wearing gloves? | 1, Always, as recommended  2, Most of the time  3, Occasionally  4, Rarely |
| Q20 | During a health care interaction with the COVID-19 patient, were high touch surfaces decontaminated frequently (at least three times daily)? | 1, Always, as recommended  2, Most of the time  3, Occasionally  4, Rarely |
|  |  |  |
|  | **Section D: Adherence to IPC measures when performing aerosol-generating procedures (e.g. tracheal intubation, nebulizer treatment, open airway suctioning, collection of sputum, tracheotomy, bronchoscopy, cardiopulmonary resuscitation (CPR), etc.).** | |
|  | *For the following questions, please quantify the frequency with which you wore PPE, as recommended: ‘Always, as recommended’ means more than 95% of the time; ‘Most of the time’ means 50% or more but not 100%; ‘occasionally’ means 20% to under 50% and ‘Rarely’ means less than 20%.* | |
| Q21 | During aerosol-generating procedures on a COVID-19 patient, did you wear personal protective equipment (PPE)? | 1, Yes  2, No |
|  | **If yes, for each item of PPE below, indicate how often you used it?** |  |
|  | Single-use gloves | 1, Always, as recommended  2, Most of the time  3 Occasionally  4, Rarely |
|  | N95 mask (or equivalent respirator) | 1, Always, as recommended  2, Most of the time  3, Occasionally  4, Rarely |
|  | Face shield or goggles/protective glasses | 1, Always, as recommended  2, Most of the time  3, Occasionally  4, Rarely |
|  | Disposable gown | 1, Always, as recommended  2, Most of the time  3, Occasionally  4, Rarely |
| Q22 | During aerosol-generating procedures on the COVID-19 patient, did you remove and replace your PPE according to protocol (e.g., when medical mask became wet, disposed the wet PPE in the waste bin, performed hand  hygiene, etc.)? | 1, Always, as recommended  2, Most of the time  3 Occasionally  4, Rarely |
| Q23 | During aerosol-generating procedures on the COVID-19 patient, did you perform hand hygiene before and after touching the COVID-19 patient, regardless of whether you were wearing gloves? | 1, Always, as recommended  2, Most of the time  3 Occasionally  4, Rarely |
| Q24 | During aerosol-generating procedures on the COVID-19 patient, did you perform hand hygiene before and after any clean or aseptic procedure was performed? | 1, Always, as recommended  2, Most of the time  3 Occasionally  4, Rarely |
| Q25 | During aerosol-generating procedures on the COVID-19 patient, did you perform hand hygiene after touching the patient’s surroundings (bed, door handle, etc), regardless of whether you were wearing gloves? | 1, Always, as recommended  2, Most of the time  3 Occasionally  4, Rarely |
| Q26 | During aerosol-generating procedures on the COVID-19 patient, were high-touch surfaces decontaminated frequently (at least three times daily)? | 1, Always, as recommended  2, Most of the time  3 Occasionally  4, Rarely |
