# Supplementary material for: Infection prevention and control compliance among exposed healthcare workers in COVID-19 treatment centers in Ghana: A descriptive cross-sectional study
Source: PLoS One. 2021 Mar 9;16(3):e0248282. doi: 10.1371/journal.pone.0248282 (PMC7943010; doi:10.1371/journal.pone.0248282)
Supplement: Attachment — Submitted filename: Response to reviewers.docx [file pone.0248282.s004.docx]

10^th^ February, 2021

**To PLOS ONE Academic Editor (Wen-Jun Tu)**

**From the corresponding author (Dr. Mary Eyram Ashinyo)**

First of all, We would like to express my sincere gratitude to you for devoting part of your schedules for our manuscript and for the valuable comments. We also express our profound gratitude to the reviewers for devoting their golden time to review this manuscript.

Below are our point by point responses to the comments raised during the review process.

1. Please ensure that your manuscript meets PLOS ONE's style requirements, including those for file naming. The PLOS ONE style templates can be found at

<https://journals.plos.org/plosone/s/file?id=wjVg/PLOSOne_formatting_sample_main_body.pdf> and

<https://journals.plos.org/plosone/s/file?id=ba62/PLOSOne_formatting_sample_title_authors_affiliations.pdf>

**Response 1: we accept this comment and we have made changes to reflect PLOS ONE style requirements**

2. Thank you for stating in the text of your manuscript "The study was part of a large study titled “Exposure risk Assessment: A survey among frontline healthcare workers in designated COVID-19 treatment centers”, which was approved by the Ghana Health Service Ethics Review Committee. Written informed consent was obtained from all study participants.". Please also add this information to your ethics statement in the online submission form.

**Response 2: We accept this comment and we have incorporated in the online submission form**

3. Please include additional information regarding the survey or questionnaire used in the study and ensure that you have provided sufficient details that others could replicate the analyses. For instance, if you developed a questionnaire as part of this study and it is not under a copyright more restrictive than CC-BY, please include a copy, in both the original language and English, as Supporting Information.

**Response 3: We accept this comment and we have incorporated the survey questionnaire as additional file “S1 File”**

4. We noted in your submission details that a portion of your manuscript may have been presented or published elsewhere. ("Yes, It does not constitute a dual publication because the pending manuscripts exposure level of healthcare workers to the COVID-19 virus while the current manuscripts went ahead to measured  IPC compliance among healthcare workers who were exposed to a COVID-19 patients.) Please clarify whether this conference proceeding or publication was peer-reviewed and formally published. If this work was previously peer-reviewed and published, in the cover letter please provide the reason that this work does not constitute dual publication and should be included in the current manuscript.

**Response 4: We have provided the reasons for which this manuscript should not be considered as a dual publication in the cover letter. We have also included the related manuscript in this current manuscript.**

5. Please ensure that you refer to Figure 3 in your text as, if accepted, production will need this reference to link the reader to the figure.

**Response 5: We accept this comment and figure 3 have been cited appropriately in the text**

6. We note you have included tables to which you do not refer in the text of your manuscript. Please ensure that you refer to Tables 3 and 4 in your text; if accepted, production will need this reference to link the reader to the Table.

**Response 6: We accept this comment and Table 3 and 4 have been cited accordingly in the text.**

7. Please include captions for your Supporting Information files at the end of your manuscript, and update any in-text citations to match accordingly. Please see our Supporting Information guidelines for more information: http://journals.plos.org/plosone/s/supporting-information.

**Response 7:  We accept this comment. Captions for supporting files have been updated and cited accordingly**

 Lines 96-98: you wrote "The sample size was calculated using the Cochran formula at a 95% confidence interval, an assumed exposure level of 50%, and a 5% margin of error."

Please quote the source, and include it in the References.
If the Cochran formula is not too long, include it as well.

**Response 8 : We accept this comment and we have reference it appropriately. We have also included the Cochran formula.**

- Line 117: "occasionally"

You forgot to close the quotation marks.

**Response 9: We accept this comment and quotation mark have been added accordingly**

- Lines 306-307: "Maliszewska M, Mattoo A, Van Der Mensbrugghe D. The potential impact of COVID-19 on GDP and trade: A preliminary assessment"
Please include the year of the publication

**Response 10: The year of publication have been incorporated appropriately.**
