# Supplementary material for: Infection prevention and control compliance among exposed healthcare workers in COVID-19 treatment centers in Ghana: A descriptive cross-sectional study
Source: PLoS One. 2021 Mar 9;16(3):e0248282. doi: 10.1371/journal.pone.0248282 (PMC7943010; doi:10.1371/journal.pone.0248282)
Supplement: Attachment — Submitted filename: Response to reviewers.docx.docx [file pone.0248282.s005.docx]

19^th^ February, 2021

**To PLOS ONE Academic Editor (Wen-Jun Tu)**

**From the corresponding author (Dr. Mary Eyram Ashinyo)**

First of all, We would like to express my sincere gratitude to you for devoting part of your schedules for our manuscript and for the valuable comments. We also express our profound gratitude to the reviewers for devoting their golden time to review this manuscript in the second round.

Thank you.

Comments and **responses**

1. In order to provide a more complete information to our readers on the topic, we would like to emphasize the importance to cross referencing very recent material on the same topic published in "PLoS ONE ". Therefore, it would be highly appreciated if you would check the contents published in the last two years of "PLoS ONE" (<https://journals.plos.org/plosone/>) and add all material relevant to your article to the reference list.

**We accept this recommendation, but we are unable to add references that cannot be cited in the text. We have however, added articles published in PLOS ONE which are citable in our work. References 9, 11, 13, 32, 34 and 36**
2. Add “Clinical Features and Short-term Outcomes of 102 Patients with Corona Virus Disease 2019 in Wuhan, China. Clinical Infectious Diseases, 71(15):748-755” in the revision text。

**We accept this recommendation and we have cited it in the manuscript, reference 6**
3. The grammar needs to be edited throughout.

**The manuscript have undergone grammar editing**
Reviewer #1: Lines 78-80:
Please, mention Covid-19 vaccines as well. For example, here you can find some clues:
- Matrajt et al., 2021. Vaccine optimization for COVID-19: Who to vaccinate first? Science Advances Vol. 7, no. 6, eabf1374. DOI: 10.1126/sciadv.abf1374

**We accept this comment and we have incorporated it accordingly. Reference 16; lines 83-84**

Line 93: “formula [12];” and “with the following parameters;”
You should use “:” instead of “;”.
**we have modified it to suit this comment, but we rather used “at” 95% confidence interval (1.96)**

Lines 258-169: I did not understand how you obtained 23.2% for “PPE use total”. Could you please recheck it and clarify it in the text? Because it seems very low, compared to the other individual values you showed, and even checking Supporting information – S1 file and S1 table, it is not clear to me how you got that value. Therefore, a better explanation would be needed.

**Thank you very much for this important comment. We realized that there was a mistake in recoding some of the variables into compliant and non-compliant. This has been rectify accordingly. How compliant and non-compliant was measure have been explained in Table 1 and 2.**
